# Consumption Patterns of Fruit and Vegetable Juices and Dietary Nutrient Density among French Children and Adults

**DOI:** 10.3390/nu7085268

**Published:** 2015-07-23

**Authors:** Aurée Francou, Pascale Hebel, Véronique Braesco, Adam Drewnowski

**Affiliations:** 1 CREDOC, Centre de Recherche pour l’EtuDe et l’Observation des Conditions de vie, 142 rue du Chevaleret, Paris 75013, France; E-Mails: francou@credoc.fr (A.F.); hebel@credoc.fr (P.H.); 2 VAB-nutrition, 1 rue Claude Danziger, Clermont-Ferrand 63100, France; E-Mail: veronique.braesco@vab-nutrition.com; 3 Center for Public Health Nutrition, University of Washington, P.O.Box 353410, Seattle, WA 98195, USA

**Keywords:** fruit, vegetable, nutrient density, consumption patterns

## Abstract

Background: Fruit and vegetable consumption is a marker of higher-quality diets; less is known about the contribution of 100% fruit and vegetable juices (FVJ) to diet quality. Objective: To explore FVJ consumption patterns in relation to dietary nutrient density among French children (aged 3–14 years old) and adults (≥21 years old). Methods: Analyses were based on the nationally representative 2013 CCAF (Comportements et Consommations Alimentaires en France) survey of 1930 respondents, stratified by age group, FVJ consumption, and socioeconomic status (SES). Dietary nutrient density was based on the Nutrient Rich Food (NRF9.3) index, adjusted for gender and age. Results: Mean total consumption of fruits and vegetables was 2.6 servings/day for children and 3.8 servings/day for adults. Mean population consumption of FVJ was 83 mL/day for children and 54.6 mL/day for adults, equivalent to 0.4 servings/day and 0.3 servings/day respectively. FVJ consumers had higher quality diets than did non-consumers, after adjusting for covariates. The respective NRF9.3 values were 486.4 ± 4.3 *vs*. 428.7 ± 7.5 for children and 460.7 ± 4.4 *vs.* 435.4 ± 4.4 for adults. FVJ consumers had similar or higher intakes of fruits and vegetables than did non-consumers. The socioeconomic gradient for FVJ consumption was much weaker (*p* < 0.046) than for whole fruit (*p* < 0.01). Conclusions: In a nationally representative sample of French children and adults, fruit and vegetable consumption fell short of recommended values. Higher FVJ consumption was associated with higher-quality diets and better compliance with the French National Plan for Nutrition and Health (PNNS).

## 1. Introduction

Fruit and vegetable consumption in France and in the US falls well below the national Five-A-Day goals [1,2,3]. The What We Eat in America survey estimated fruit consumption in the US at only 1.1 daily servings and vegetable consumption at 1.4 daily servings [4]. Based on the 2006 national food consumption data for France, 73% of adults and 95% of children failed to consume five daily servings of fruits and vegetables as recommended by the French National Plan for Nutrition and Health [5]. In general, least likely to meet the Five-A-Day goals were groups of lower socioeconomic status (SES) [6,7,8,9,10] and those living in disadvantaged areas [11,12].

The consumption of 100% fruit juices, along with whole fruit, is one way to meet total fruit consumption goals [13]. In the US, the inclusion of 100% juice in dietary patterns did not appear to displace whole fruit. Recent analyses showed that whole fruit contributed fully two thirds to total fruit consumption, with only one third coming from 100% juice [13]. However, whereas whole fruit consumption was highest among older adults and among groups with higher education and incomes, no social gradient was observed for 100% fruit juice. Fruit juice consumption was highest among children and declined sharply with age [13].

In the US, one cup of fruit and vegetable juices (FVJ) (224 mL) is considered to provide one serving from the fruit or vegetable group [3]. The 2008 French dietary guidelines specify that up to one of the five daily fruit and vegetable servings can be in the form of FVJ [14]. FVJ can contribute to meeting dietary guidelines for children in France, when limited to 100–200 mL/day (½ to one cup per day) [15]. The FVJ in France are mostly fruit juices, while the consumption of vegetable juices (mostly tomato) is very low.

This study used the nationally representative 2013 CCAF database [16] to examine the impact of FVJ consumption on dietary nutrient density in France. FVJ consumption patterns were stratified by age group and by SES. The Nutrient Rich Food (NRF9.3) index, a published and validated nutrient profiling model [17], adjusted for age and gender, was the principal measure of diet nutrient density. The hypothesis was that higher FVJ consumption in France would be independently associated with higher-quality diets, after adjusting for demographic and lifestyle covariates.

## 2. Materials and Methods

### 2.1. CCAF Participants

Dietary intake data were based on the nationally representative 2013 Comportements et Consommations Alimentaires en France (CCAF) dietary survey that was conducted by CREDOC (Centre de Recherche pour l’ÉtuDe et l’Observation des Conditions de vie, Paris, France), between October 2012 and July 2013. The quota survey sampling method took into account the geographical region, town size, participant age, household size, and socio-economic status, based on the 1999 French census. Details of participant recruitment have been published [16].

The survey was carried out in a national representative sample of 1250 French households resident in metropolitan France. Metropolitan France refers to France itself, as opposed to overseas possessions and territories. Children were oversampled to give a national representative sample of 1000 children 3–17 years old. The age distribution in the 2013 CCAF was 809 children (3–14 years old); 109 adolescents (15–20 years old), and 1121 adults (≥21 years old). The present analyses were based on children (3−14 years old, *n* = 809) and adults (≥21 years old, *n* = 1121). The adolescents were omitted as providing insufficient power for analysis. The CCAF study had been approved by the institutional review board for human subjects research and subjects provided informed consent [16].

Age, body weight, and height were self-reported during an in person interview. Body mass index (BMI: kg/m^2^) values were calculated. For adults, normal weight was defined as BMI: 18.5–25 kg/m^2^; overweight as BMI: 25–30 kg/m^2^ and obesity as BMI > 30 kg/m^2^. In children, overweight was defined based on growth curves and standard cut-off values [18].

### 2.2. Dietary Intake Data

Dietary intake data were based on a seven-day dietary history interview for all household members aged 3 years old and above. For children aged <9 years old, dietary information was provided by parents or caregivers. The participants (adults and children >9 years old) completed a seven-day food survey, where respondents reported the types and amounts of all food and beverages consumed in the preceding 24-h. The participants were provided with the SUVIMAX portion size atlas, used and validated in previous research [19] that provided photographs of food portions along with 7 potential response options. The portion size atlas was provided in the paper format (54%) and online (46%).

Reported energy intakes were compared to the estimated energy requirements (1.55 times the metabolic rate) for age, body weight and height [20]. Adult participants were excluded if their reported intake was inconsistent with the estimated energy requirements [16]. Children were excluded if their total energy intakes divided by estimated basal metabolic rate was ≥0.05.

### 2.3. Energy and Nutrient Intakes

Energy and nutrient intakes were estimated using the 2013 food composition table developed by the French agency for Food, Environmental and Occupational Health & Safety (ANSES). Usual intakes of total fruit (whole fresh fruit + cooked fruit + fruit juice), whole fruit, and 100% FVJ were assessed for the entire population and for population subgroups. The term “100% FVJ” covers fruit and vegetable juices NFC (not from concentrate) and FC (from concentrate). The 2013 CIQUAL database provided nutrient values for the principal 100% FVJ juices consumed: orange, apple, grape, grapefruit, pineapple, and multi-fruit and exotic fruit (mango, coconut) as well as tomato, carrot, and multi-vegetable juice. Nectars and juice-based soft drinks were excluded. Under European and French regulations, the addition of sugars to 100% FVJ is prohibited [21].

Although FVJ cannot contain added sugars, they do contain naturally present “free” sugars. The World Health Organization (WHO) defines free sugars [22] as “all monosaccharides and disaccharides added to foods by the manufacturer, cook, or consumer, plus sugars naturally present in honey, syrups, and fruit juices”. The values for added sugars and free sugars in the CIQUAL database were obtained from ANSES; from published nutrient composition data, and from the nationwide online study Nutrinet Santé [23].

### 2.4. Compliance with the French National Program on Nutrition and Health

The National Program on Nutrition and Health (Programme National Nutrition Santé—PNNS) was implemented under the Ministry of Health in France in 2001 and is regularly updated. Consuming at least 5 serving of fruits and vegetables per day is one of the 9 key public health objectives. Included in the recommendation are all fruits and vegetables, fruit or vegetable juices (FVJs), and mixed foods that contain fruits and vegetables. Serving sizes were defined as 80 g for vegetables, 200 g for soups, 80 g for fruits and 200 mL for FVJ (with a maximum of one serving per day). The degree of compliance with the PNNS was assessed for each CCAF participant.

### 2.5. Dietary Nutrient Density Measure

The Nutrient Rich Foods (NRF) index, a published and validated nutrient profiling model [24] was the principal measure of dietary nutrient density. The NRF9.3 nutrient profiling model was based on the sum of percent daily values (%DVs) for nine nutrients to encourage, minus the sum of percent daily values for 3 nutrients to limit. Percent DVs were calculated based on the total diet. The nutrients to encourage were protein, fiber, vitamins A, C and E, calcium, iron, potassium and magnesium. The negative LIMiting nutrients (LIM) subscore was based on maximum recommended values (MRVs) for saturated fat, free sugars, and sodium [17,25]. In essence, nutrient profile models applied to individual foods calculate the ratio of nutrients to calories per reference amount. The same profiling algorithms can be applied to individual foods, composite meals, or the total diet [26]. In order to apply the NRF9.3 algorithm to the assessment of diets, as opposed to individual foods, it was necessary to adjust the dietary reference values for gender and age. Nutrient profile models designed to evaluate individual foods are typically based on a single set of nutrient standards. In the US, such standards are based on Food and Drug Administration (FDA) regulatory and labeling requirements and are calculated per reference amount of food (serving size).

By contrast, dietary reference values as applied to total diets are typically adjusted for gender and age. Both the Reference Daily Intakes (RDIs) in the US and the Recommended Dietary Intakes (Apports Nutritionnels Conseillés or ANC) in France are both age- and gender-specific. A summary of NRF index nutrients and their ANC values by age and gender is provided in Table 1.

In this set of analyses, NRF9.3 values for each participant were based on age- and gender-adjusted ANC values for the nine qualifying nutrients. Free sugars, saturated fats, and sodium were expressed as percent MRVs. In this manner, an age- and gender-appropriate nutrient density score was calculated for each CCAF participant.

**Table 1:** Recommended dietary amounts (Apports Nutritionnels Conseilles or ANC) in France by age and gender for use with nutrient rich food (NRF) scores.

Subscore	Nutrient	Age Group (Years Old)																													
		3		4–6		7–9		10		11–12		13–14		15		16–17		18–19			20–55		56–65			66–74			>75		
**NR 9**	Proteins (g/kg bwt)	**Details are described below**																													
	Fibers (g)	14				16				19				21				25													
	Vitamin A (IU)	400		450		500		550				Male: 700 Female: 600				Male: 800 Female: 600															
	Vitamin C (mg)	60		75		90		100				110																		120	
	Vitamin E (mg)	6		7,5		9		11				12																		20	
	Calcium (mg)	500		700		900		1200													900		Male: 900 Female: 1200			1200					
	Iron (mg)	7				8		10				Male: 13 Female: 16									Male: 9 Female: 16		9							10	
	Potassium (mg/kg bwt)	40																													
	Magnesium (mg)	80		130		200		280				Male: 410 Female: 370									Male: 420 Female: 360									400	
**LIM**	Saturated fat (g)	12% of total energy intake (ANSES)																													
	Free sugar (g)	10% of total energy intake (OMS)																													
	Sodium (mg)	Children and pregnant female: 2600 mg/day, male: 3200 mg/day (ANSES)																													
**Proteins (g/kg bwt) in NR 9**																															
Age (Years Old)			3		4		5		6		7		8		9		10		11	12		13		14	15		16	17			>18
Male			0.90		0.86		0.85		0.89		0.91		0.92		0.92		0.91		0.91	0.90		0.90		0.89	0.88		0.87	0.86			0.83
Female																			0.90	0.89		0.88		0.87	0.85		0.84	0.83			

### 2.6. Physical Activity and Screen Time

CCAF participants reported the time spent on watching various screens (television, computer, video games, *etc.*) and the time spent on physical activities. The screen time variable was dichotomized for adults (<3 h/day or >3 h/day) and for children (<2 h/day or >2 h/day) based on distribution of the data. The physical activity variable was also dichotomized. For adults, the time spent on physical activities such as household activities, gardening, sports, *etc*., was <2 h/day or >2 h/day. For children, the time spent on sports was <4 h/week or >4 h/week.

### 2.7. Statistical Analyses

FVJ consumers were defined as those CCAF participants who listed FVJ at least once in the seven-day diet history. Analyzes of FVJ consumption were conducted separately for children and adults. Additional strata were based on gender (male, female) and socioeconomic status (SES), as assessed by occupation or education of head of household. Data were reported as means and standard errors and/ or medians and percentiles. The statistical significance level was set at *p* < 0.05.

Differences in dietary nutrient density between FVJ consumers and non-consumers of FVJ were tested using the generalized linear model (PROC GLM). Quartile analysis of NRF scores, based on amounts of FVJ consumption was also carried out.

Multiple regression was carried out to explore potential association between amounts of FVJ consumed and NRF9.3 scores, adjusting for FVJ consumption (yes, no), age, gender (for adults only), education, and screen time. The SAS 9.2 software was used for statistical analyses (SAS Institute, Inc., Cary, NC, USA).

## 3. Results

Children consumed 279 g/day of total fruit and vegetables on the average, corresponding to 2.6 servings/day. Adults consumed 392 g/day, corresponding to 3.8 servings/day. On the average, 94.9% of all children and 73.0% of adults failed to meet the PNNS fruit and vegetable goals.

These age trends were reversed for FVJ consumption, consistent with data from the US [13]. Children aged 3–14 years old consumed 83 mL/day FVJ whereas adults aged >21 years old consumed 54.6 mL/day. On per weight basis, FVJ accounted for about 30% of total fruit and vegetable intakes (in g) among children but only 14% of fruit and vegetable intakes among adults.

Assuming a 200 mL FVJ serving size, FVJ consumption was 0.4 servings/day for children and 0.3 servings/day for adults. Nine out of ten children (89.8%) and adults (93.3%) consumed less than one serving (200 mL) of FVJ per day. The consumption of vegetable juices was very small: 0.1 mL/day for children and 0.9 mL/day for adults.

The proportion of FVJ consumers in the population was estimated at 71.1% of children but only 48.6% of adults. The proportion of vegetable juice consumers was extremely low (<1%). Among FVJ consumers, median FVJ consumption was 102.9 mL/day for children and 91.4 mL/day for adults. Significantly, those children who consumed FVJ also consumed more fresh fruit as compared to non-consumers (65.2 ± 2.8 g/day *vs*. 41.7 ± 3.2 g/day). The consumption of cooked fruit (“compote”) was also higher among FVJ consumers as compared to non-consumers for both children and adults.

**Figure 1:**
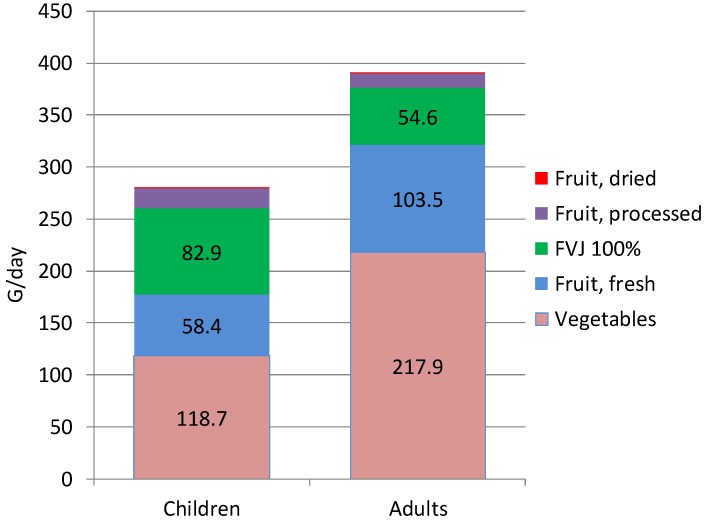
Contribution of vegetables, whole fruit, and FVJ (fruit and vegetable juices) to total fruit and vegetable consumption of children and adults (in g/day).

Figure 1 shows mean total consumption of fruit and vegetables (in g/day) for children and adults. It can be seen that fruit, fresh and processed and FVJ each provided about 30% of total fruit and vegetables intakes among children. Among adults, the contribution of whole fruit remained at 30% but the contribution of FVJ dropped to 14%.

Persons identified as FVJ consumers consumed more total servings of fruit and vegetables per day. Among children, the difference was 0.9 additional servings (from 1.9 servings to 2.8 servings), whereas among adults, the difference was 0.7 servings (from 3.5 servings to 4.2 servings). Among both children and adults, FVJ consumers were more likely to reach the Five-A-Day target than were non consumers (children: 6.7% *versus* 1.3%; adults: 31.2% *versus* 23.0%). However, no differences in total vegetable intakes were observed for FVJ consumers *vs.* non-consumers.

Subsequent analyses compared dietary patterns and diet quality measures of FVJ consumers and non-consumers. Table 2 shows differences between FVJ consumers and non-consumers in total NRF9.3 nutrient density score, its components (NR and LIM), and individual nutrients. Using free (as opposed to added) sugars to calculate the LIM components of the NRF score was consistent with the current policies of the WHO. Calculations of the LIM and NRF scores were also performed using added sugars.

The data are presented separately for children and adults. As noted above, percent daily values and NRF scores calculated for each CCAF participant were adjusted for age and gender. FVJ consumers had significantly higher total NRF9.3 scores than did non-consumers.

Nutrient-by-nutrient analyses showed that FVJ consumers had diets that were significantly higher in vitamin C, vitamin E, calcium and potassium compared to non-consumers. Among children, FVJ consumers had diets that were also higher in fiber, vitamin A, and magnesium, as compared to diets of non-consumers. FVJ consumption was not associated with differences in saturated fat but the diets of FVJ consumers were higher in sodium and free sugars.

**Table 2:** NRF9.3 dietary nutrient density scores, subscores and individual components by juice consumers *versus* non-consumers for each age group.

	Children (3–14 years old)			Adults (≥21 years old)		
	Consumers *N* = 575	Non-Consumers *N* = 234	*p*	Consumers *N* = 544	Non-Consumers *N* = 577	*p*
**NRF9.3 score**	481.3 ± 4.4	428.7 ± 7.5	0.0000	460.7 ± 4.4	435.4 ± 4.4	0.0000
Protein (g/kg)	99.5 ± 0.2	99.5 ± 0.3	0.9384	99.1 ± 0.2	99.3 ± 0.1	0.2259
Fiber	81.0 ± 0.8	75.0 ± 1.3	0.0001	69.4 ± 0.8	68.5 ± 0.8	0.4384
Vitamin A	78.6 ± 1.0	73.4 ± 1.7	0.0067	81.4 ± 1.0	79.8 ± 1.0	0.2634
Vitamin C	79.7 ± 1.0	47.6 ± 1.6	0.0000	80.5 ± 1.0	56.4 ± 1.2	0.0000
Vitamin E	63.9 ± 0.9	55.9 ± 1.5	0.0000	61.5 ± 1.0	54.9 ± 1.0	0.0000
Calcium	81.8 ± 0.9	74.7 ± 1.6	0.0000	85.8 ± 0.8	81.3 ± 0.8	0.0001
Iron	87.9 ± 0.7	85.5 ± 1.2	0.0967	85.7 ± 0.9	87.4 ± 0.8	0.1693
Potassium	98.0 ± 0.3	96.0 ± 0.7	0.0032	91.7 ± 0.5	89.4 ± 0.6	0.0034
Magnesium	88.8 ± 0.7	83.3 ± 1.4	0.0001	78.0 ± 0.7	76.2 ± 0.7	0.0759
**Nutrient Rich subscore**	759.1 ± 4.7	690.8 ± 8.2	0.0000	733.1 ± 4.4	693.1 ± 4.3	0.0000
Saturated fat	97.2 ± 0.3	97.6 ± 0.4	0.4491	96.5 ± 0.4	96.4 ± 0.4	0.8847
Sodium	83.0 ± 0.8	73.5 ± 1.3	0.0000	93.7 ± 0.5	91.8 ± 0.5	0.0090
Free sugars	97.6 ± 0.3	91.0 ± 1.2	0.0000	82.1 ± 1.1	69.5 ± 1.2	0.0000
**LIMiting subscore**	277.8 ± 0.9	262.1 ± 1.8	0.0000	272.3 ± 1.2	257.7 ± 1.3	0.0000

Data are expressed in terms of age- and gender specific percent daily values for each nutrient; presented are means and standard errors of the mean (SEMs).

**Figure 2:**
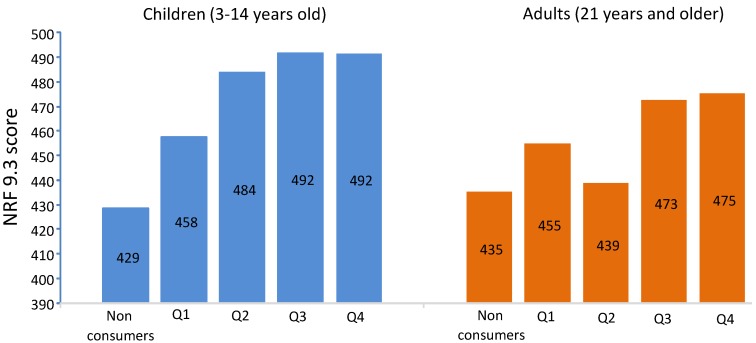
NRF9.3 score values (y axis) for FVJ (fruit and vegetable juices) non consumers and for consumers stratified by quartiles of consumption. Data are presented for children and for adults. The NRF9.3 score was calculated using free sugars in the LIM subscore.

Figure 2 shows a positive dose-response relation between quartiles of FVJ intake and NRF values. Mean FVJ intakes from Q1 to Q4 for children were 29 mL/day, 72 mL/day, 129 mL/day and 240 mL/day. Mean FVJ intakes from Q1 to Q4 for adults were 23 mL/day, 65 mL/day, 118 mL/day and 247 mL/day. The increase in NRF as a function of FVJ consumption was significant for both children and for adults. The NRF9.3 score was based on free as opposed to added sugars. Comparable results were obtained when added sugars were used in calculating the LIM score (data not shown).

**Figure 3:**
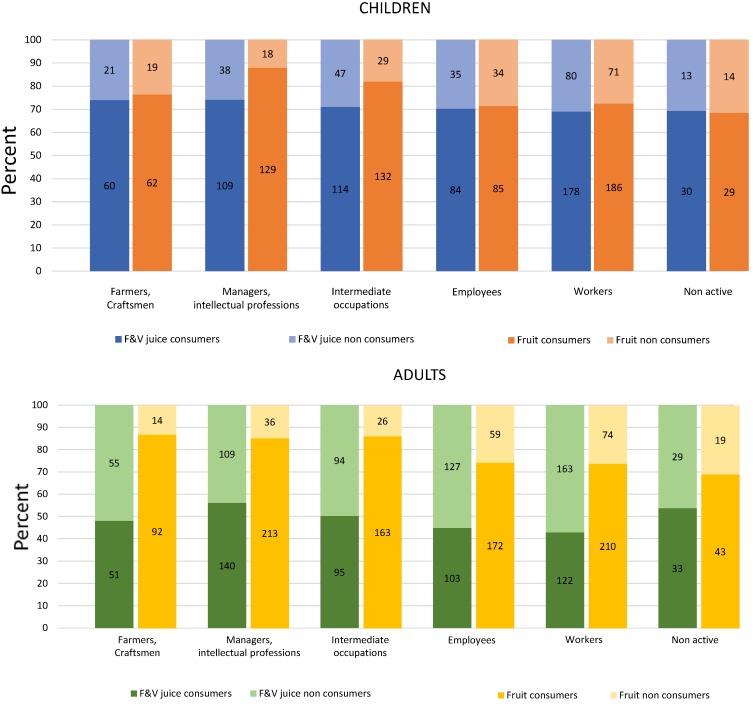
Whole fruit and FVJ (fruit and vegetable juices) consumption by occupation of health of household. Data are for children (3–14 years old) in the top panel and for adults (≥21 years old) in the bottom panel. Figures in the histogram bars represent the number of subjects in each category.

Figure 3 shows FVJ *versus* whole fruit consumption by occupational status of head of household, a measure of SES. Whole fruit consumption was associated with the educated and professional classes; the social gradient held for both children (*p* = 0.0018) and adults (*p* < 0.0001). By contrast, FVJ consumption showed less of a social gradient (significance for adults only: *p* = 0.0435). Among children, whole fruit consumers were also likely to be of higher SES; the strength of the SES gradient was reduced among FVJ consumers. Similarly, higher SES adults were more likely to consume whole fruit; the social gradient in FVJ consumption was attenuated.

Multivariable analyses of FVJ consumption in relation to NRF9.3 diet quality scores are summarized in Table 3. There was a significant relation between higher FVJ consumption and diet quality (Model 1) that held after adjusting for socio-demographic covariates. Higher NRF scores were associated with the younger age group; children had higher quality diets than did adults. The NRF measure of diet quality was also higher for men than for women; and increased with education and less sedentary lifestyles, as captured by screen time.

**Figure 4:**
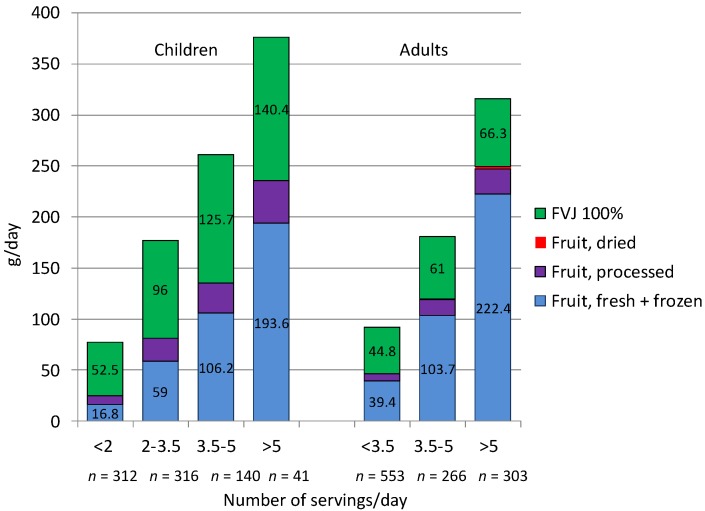
Contribution of fruit and FVJ (fruit and vegetable juices) to total fruit intakes by PNNS (Programme National Nutrition Santé) level of consumption.

Figure 4 shows how greater compliance with the French PNNS dietary guidelines was reflected in higher consumption of whole fruit and higher consumption of FVJ. Categories on the *x*-axis represent the number of fruit and vegetable servings, up to and exceeding Five-A-Day. As might be expected, only a minority of respondents met the Five-A-Day goal, especially among children. The number of respondents in each category is indicated on the *x*-axis.

Whole fruit and FVJ consumption, shown on the *y*-axis, is in g per day. It can be seen that whole fruit accounted for most of total fruit consumption and that FVJ did not displace whole fruit from the diet.

**Table 3:** Multivariable regressions to assess the relation between FVJ (fruit and vegetable juices) consumption and NRF9.3 scores after adjusting for socio-demographic and lifestyle covariates. Data are presented as means and confidence intervals.

	Model 1				Model 2			
	B	CI		*p*	B	CI		*p*
**NRF9.3 scores**	436.6	(376.0	497.2)	<0.0001	501.1	(441.1	561.0)	<0.0001
**Change in NRF9.3 score for FVJ consumers *vs*. non-consumers**								
**Consumers**	+39.7	(30.0	49.4)	<0.0001	+33.7	(24.0	43.4)	<0.0001

Model 1: Adjusted for region, city, household size, household type; Model 2: Model 1 + adjusted for age, gender, education, screen time.

## 4. Discussion

The present analyses, based on a large nationally representative sample of French children (ages 3–14 years old) and adults aged ≥21 years old, confirmed that fruit and vegetable consumption was well below the national guidelines. About 94.9% of children and 73.0% of adults failed to meet the national fruit and vegetable guidelines of five servings per day. Prior analyses of national consumption trends between 1999 and 2007 showed that vegetable consumption declined while total fruit consumption was stable. An increase in consumption was observed only among older adults (>55 years old) [27].

According to the 2008 French guidelines, FVJ can supply up to one of the five daily fruit and vegetable servings. However, the current population consumption means for FVJ were far below these modest norms. Mean FVJ consumption was estimated at only 0.4 daily servings for children and 0.3 servings for adults. The data also showed that almost 30% of children and 50% of adults did not consume any FVJ at all. Only one in 10 children and one in 14 adults consumed one glass (200 mL) or more of FVJ per day.

These shortfalls in fruit and in FVJ consumption were larger than expected. However, the French data were largely consistent with previously reported results for the US [13], there too, whole fruit provided about 65% of total fruit consumption, while 100% juice provided about 35%. Whereas whole fruit consumption was higher among those with higher incomes or more education, juice was more likely to be consumed by children, minorities and lower-income groups.

In both countries, FVJ consumption was well below the recommended norms. The 2010 Dietary Guidelines for Americans have recommended that no more than half of fruit servings be from 100% juice, while others have recommended limiting 100% fruit juice to one or two servings per day [3]. In France, one of the five fruit and vegetable servings can be supplied by juice. The present results are consistent with the US data in showing that juice did not displace whole fruit from the diet [13].

The implications of the study of policy and practice are therefore clear: the dramatic shortfalls in fruit consumption can be met by a combination of fruit and juice, with the relative proportions of whole fruit and juice following established guidelines and norms. In the US, such norms have been proposed by the American Academy of Pediatrics [13]; in France, the Five-A-Day guideline is proposed by the National Plan for Nutrition and Health.

Both the French and the US data have identified a socioeconomic component in the consumption of whole fruit that was not observed with 100% juice [13]. This is an important finding since the total fruit goals in the US were least likely to be met by groups of lower education and incomes [6,7]. Based on analyses of the nationally representative National Health and Nutrition Examination Survey (NHANES), it would appear that the more affordable and accessible 100% juices might represent a point of entry to the fruit and vegetable category for lower-income groups. Preliminary analysis using French national statistics (INSEE) suggest that one portion of FVJ could be obtained in France for about 60% of the price of a portion of the corresponding whole fruit [24].

Two more points deserve mention. First, FVJ did not displace whole fruit from the diet. The PNNS goals were met by a combination of whole fruit and FVJ. Second, FVJ consumers appeared to have higher quality diets; the NRF scores for FVJ consumers were higher than for non-consumers. Calculations of the NRF score were based on free rather than added sugars. Given that FVJ cannot contain added sugars but do contain naturally occurring free sugars, such a base of calculation might be expected to be detrimental to juices. However, the data showed that NRF scores were consistently higher among FVJ consumers than non-consumers, regardless of whether NRF scores were calculated using added or free sugars.

FVJ consumption was also linked to higher NRF scores in a dose dependent manner. These observations held for children and for adults. The relation was largely driven by vitamin C, although other NRF index nutrients also contributed.

The study had some limitations. First, dietary intake data for younger children in the CCAF were provided by the caregiver. Second, the sample was insufficient to permit detailed analyses of whole fruit and FVJ consumption by age group and SES. Third, data on household SES were provided by self-reports. The study also had strengths. Data of the CCAF study have been collected recently and are representative of current food intakes. The CIQUAL database was supplemented with added or free sugars, obtained from the updated Nutrient database.

## 5. Conclusion

In France, consuming FVJ was independently associated with higher-quality diets, after adjusting for covariates. FVJ consumers were more likely to meet total fruit and vegetable goals that are promoted by the French National Plan for Nutrition and Health. Higher levels of fruit consumption were achieved by a combination of whole fruit and FVJ. Given the lack of a social gradient for FVJ, the consumption of FVJ, along with whole fruit, may provide a viable mechanism to reach total fruit goals at an affordable cost.

## Abbreviations

ANSES: French National Agency for Food Safety
CCAF: Comportements et Consommations Alimentaires en France
FVJ: fruit and vegetable juices
NRF9.3: Nutrient Rich Food index
LIM: Limiting nutrients subscore
PNNS: French National Plan for Nutrition and Health; Programme National Nutrition Santé
RDIs: Reference Daily Intakes
ANC: Apports Nutritionnels Conseillés or Recommended Dietary Intakes
SES: socioeconomic status
SUVIMAX: SUpplementation en VItamines et Mineraux AntioXydants
